# A Few Observations on Health Service for Immigrants at a Primary Health Care Centre

**DOI:** 10.1155/2016/6963835

**Published:** 2016-08-03

**Authors:** Thorhildur Halldorsdottir, Halldor Jonsson, Kristjan G. Gudmundsson

**Affiliations:** ^1^Health Care Centre in Glaesibaer, 104 Reykjavík, Iceland; ^2^Reykjalundur Rehabilitation Centre, 270 Reykjavík, Iceland

## Abstract

*Objective*. Icelandic society is rapidly changing, from being an ethnically homogeneous population towards a multicultural immigrant society. In the hope of optimizing the service for immigrants at the health care centre, we decided to evaluate health care utilization by immigrants.* Methods*. As a case control study we invited all immigrants that attended the health care centre during a two-week period to participate. Paired controls of Icelanders were invited for comparison.* Results*. There were 57 immigrants, 48 females and 9 males, from 27 countries. Significantly more of the immigrant women were married, *P* < 0.001. Interpreters were needed in 21% of the consultations. The immigrants often attended the clinic and had the same diagnoses as did the nonimmigrants. The immigrants evaluated the quality of the service in Iceland as 4.3 and the service in their homeland as 1.68, *P* < 0.001.* Conclusion*. Immigrants attending a health care centre in Iceland came from all over the world, had the same diagnoses, and attended the clinic as often per annum as the nonimmigrants. Only one-fifth of them needed translators. The health and health care utilization of immigrants were similar to those of nonimmigrants.

## 1. Introduction

Providing immigrants with proper health care is a challenge, given language barriers and cultural differences. At the primary health care level, it becomes the responsibility of the clinic to arrange such practical issues as providing capable interpreters when needed [1]. One of the fundamental objectives of public health care systems with universal health coverage is to achieve equal use of the health care system and equal access to the services for equal needs, named horizontal equity [2].

From ancient times, there have been resettlements of people [3]. As a result of the constant conflicts and wars around the world, millions of people have been moving, as refugees, seeking asylum. There is also immigration in search of better living conditions, often from low-income regions [4]. Today some 9% of the inhabitants of Iceland are immigrants, the same percentage as that in Europe [1, 5]. According to studies, the immigrants are often in better health than the average for the nonimmigrant population [6, 7]. Loss of social network, isolation, and low socioeconomic status, however, can often contribute to worse health of immigrants in their new homeland [7, 8]. On the other hand, immigrants to the USA have a considerably longer life expectancy than people born in the States [9].

There are studies indicating that immigrants utilize the health care system differently from the nonimmigrants, partly because they do not have access to information about the health care system and where to attend [4, 10]. Language is the basis of all communication in health care, and it takes time for immigrants to learn a foreign language sufficiently well to communicate about their health issues. Often there is a need for interpreters [11, 12]. The health of immigrants can be affected by infections prevalent in their former geographical home, and they may have genetic variations uncommon in the new country. Many of the asylum seekers have had traumatic experiences in military conflicts where human rights have been violated [13]. The immigrants and refugees present clinical symptoms according to their ethnic background, in some cultures towards somatization [14]. The health care provider should provide medical advice and treatment with understanding and respect towards the ethnicity of the immigrant [15]. 

*The Icelandic Health Care System*. The country is an island in the North-Atlantic ocean and with a population of over 330 thousand. Though not a part of the EU, it is a part of the European Economic Area. Its welfare infrastructure is similar to those in other Scandinavian countries. During the 10 years prior to this study, there had been a rapid increase in the number of immigrants, from 3% in 1998 up to 10.4% of the population in Reykjavík in 2008 [5, 16] (see Figure 2). The primary health care system in the capital area is based on 17 health care centres, each with district responsibility, and the service is mainly state financed. There are other open access units as well, including the ER ward at the National University Hospital as well as a relatively open private specialist service in Reykjavík [17].

The Health Care Centre in Glaesibaer was opened in 2006 and is served by 5 family physicians. From the beginning, there was a focus on immigrant health, their access, and discussions on cultural aspects of health care and the use of interpreters. One of the family doctors (HJ) has been educated in international health and has a long experience of working in Africa. Many of his patients were immigrants, though other family doctors had immigrants in their registers as well.

## 2. Aims

With the intent of optimizing the service for immigrants at the health care centre, we decided to compare the health care utilization of immigrants to that of nonimmigrants. This included the diagnosis, attendance rate, and identification of any differences in family structure, as well as the need for interpreters and the immigrants' opinion of the quality of the service provided.

## 3. Methods

### 3.1. Participants

The study took place at the Health Care Centre in Glaesibaer, Reykjavík, which is a Primary Health Care Clinic with a broad spectrum of services, such as consultations with family physicians and primary health care for children and pregnant women. There is, as well, a walk-in service during the day and afternoons for the acutely sick and for minor accidents. As a case control study, we invited all immigrants to participate who attended the health care centre in over a two-week period in the autumn of 2008. For the controls we also asked the nonimmigrants to participate who were attending the same kind of service, at the same time, and were paired by age and sex. The participants signed a written informed consent.

### 3.2. Data Collection

The primary care physicians were on the outlook for immigrants in the booking registers. The medical doctors conducting the study invited all immigrants who came during the time period to participate. The same medical doctors then looked at the same time at the booking register to find a nonimmigrant control, within the same age group and of the same sex who attended the same kind of service. The participants answered a structured questionnaire about country of birth, family structure, and the length of time they had lived in Iceland, as well their attitude to the service provided. The questionnaire had been translated into English, Polish, and Thai. Interpreters were provided if the immigrants did not understand Icelandic or English. The formal questions were explained and translated to the immigrant by an interpreter as needed.

### 3.3. Measures

The participants evaluated the quality of the service on a scale from zero to five. The questions used were the same as those used in a yearly quality control survey conducted on almost all public services in Iceland [18]. A medical doctor looked at information from the health care records concerning the diagnosis and how often they and their children had attended the clinic during the previous year. The information on the children was recorded for the parent participating in the study.

### 3.4. Analysis

The SPSS software package was used. A paired* t*-test was used for paired continuous variables and the chi-square test for categorical variables. *P* was set to less than or equal to 0.05.

### 3.5. Authorization

The National Bioethics Committee in Iceland approved the study, number 08-045. The study was, as well, granted permission from The Data Protection Authority, number 2008/23.

## 4. Results

**Figure 1:**
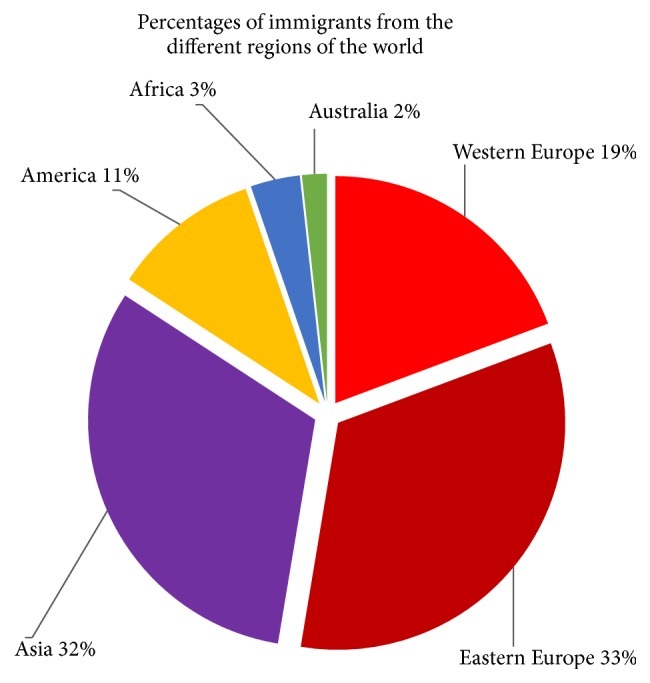


There were 57 pairs of immigrants and nonimmigrants, 48 pairs of women and 9 of men. The immigrants came from 27 countries (Table 1 and Figure 1). There were eight from Thailand and seven from Poland, five from Vietnam, four from the Philippines, and the others from different countries and continents of the world. The mean age of the participants was 34.0 years in the immigrant group and 33.7 years in the nonimmigrant group, *P* = 0.664. Of the immigrants, 26 women were married, 9 cohabiting, and 12 single, compared to 11 married, 24 cohabiting, and 13 single in the group of nonimmigrants. Of the women living in permanent relationships, being married compared to cohabiting was more common in the immigrant group, *P* < 0.001. The immigrants attended the clinic for their own consultations equally often per year as the controls or 4.6 times for the immigrant cohort and 4.7 times for the controls, *P* = 0.92. During the preceding year, the immigrants attended the clinic with their children equally often as the controls, 6.9 versus 6.6 times, *P* = 0.76 (Table 2). The immigrant group had lived in Iceland on average for 6.7 years. Interpreters were used in 12 (21%) of the 57 consultations. When asked about the quality and satisfaction with the interpreter service, the 12 immigrants rated it as 4.6, on a scale of zero to five. When asked for assessment of the quality of the service of the health care centre, the immigrants rated it as 4.3 and the controls as 4.1, *P* = 0.27. The immigrants evaluated the service in Iceland as 4.3 and the service in their homeland as 1.68, *P* < 0.001 (Table 3). There were no differences in the diagnoses of the patients between cases and controls while attending the health care centre (Table 4).

## 5. Discussion

**Figure 2:**
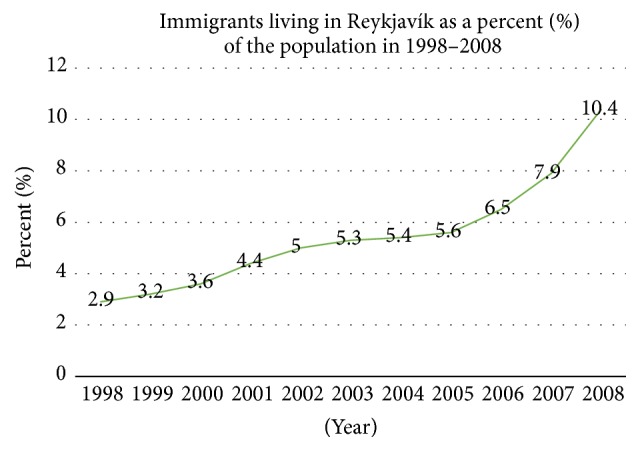


Icelandic society has been rapidly changing, from being an ethnically homogeneous population to a multicultural immigrant society. In the ten years from 1998 to 2008, the percentage of immigrants increased from 3% to 10.4% (see Figure 2) [5]. The need for good health service for immigrants has therefore become of increasing importance and a study has been needed to check on the results. To the best of our knowledge, no such similar studies on the utility of immigrant of health service have been conducted in Iceland. In fact, there are few studies on the health care utility of immigrants in clinical settings [19].

This study showed how divergent the group of immigrants in Reykjavík was, as the participants came from 27 widely separated countries. The immigrant women were more often married and the Icelandic women more often cohabiting, which was a clear cultural difference, as cohabiting has been a common form of family structure in Iceland, especially among younger people. Here we have to bear in mind that about 10.4% of the population in Reykjavík were immigrants at the time of the study and some 17% of pregnant women were of foreign origin [16]. There were no differences in the number of visits during the observation period or the preceding year, for the immigrants or nonimmigrants or their children. The participants came 4.6 times on average to the clinic, which is a similar utility rate per person as the average in Iceland, bearing in mind the sample population, many attending maternity, and child preventive services [20]. This was in contrast to a study from Sweden, where immigrants more often visited the emergency department (19% more visits) but used the outpatient clinics less often [21]. In another Swedish study from Malmö, there was hardly any difference after correcting for social and economic status [22]. In studies using a huge patient data basis from primary health care in Norway and Spain, there were fewer contacts by immigrants than nonimmigrants [23]. This is in contrast to a systematic review of the utilization of health care of immigrants in Europe, which showed that immigrants consulted general practitioners more often than nonimmigrants but used telephone contacts less frequently (perhaps because of language difficulties) [19]. Attendance at health care centres in Iceland is free of charge for children and pregnant women in preventive care, and the fee is low for other services and substantially lower than attending a hospital or private specialists. The difference in cost can cause bias, if the better-off nonimmigrants are prone to attend private specialists or hospitals. This can as well explain why immigrants use health services less often than nonimmigrants in countries without universal health care coverage. The preventive services for children and maternity care in Iceland, on the other hand, are strictly on a district basis and are the same for everyone, regardless of immigrant or nonimmigrant status or income level, and the services is not provided elsewhere in city. This was a large proportion of the cohort. The cohort is young, with obviously similar diagnosis and attendance rates.

Using an interpreter in a clinical setting is a sensitive undertaking. The doctor or the health care provider has to gather information from the patient or relatives; this information must be sufficiently clear so that it can be understood in terms of medical standards and patient needs, as well as in terms of cultural aspects [15]. Even though the immigrants had not stayed in Iceland for more than seven years on average, some 80% managed consultations on their own and spoke either Icelandic or English. Most of the participants needing an interpreter were satisfied with the interpretation.

The attitude to the quality of the service was the same among immigrants and the nonimmigrants. The results were similar to those obtained by official inquiries about the quality of the Primary Health Clinics in the capital area of Reykjavík [18]. The immigrants regarded the service provided at the Glaesibaer Public Health Care Centre as better than they were used to, a result that of course reflected the fact that a proportion of them had come from parts of the world that are low-income regions.

There were many limitations of the present study. There was only a relatively small number of participants, so it is possible that a larger study, perhaps including more than one district public health care clinic, could have detected a difference in the rate of attendance or of diagnosis. We asked all immigrants attending the clinic at the time of conducting the study to participate. However, some refused to participate and we do not have any information about their number nor their backgrounds. The present study had a high proportion of women, and this could have caused bias. The group of immigrants was young, with a mean age of 34 years, reflecting the fact that 21 of them were attending preventive care for maternity or children. We do not have any information as to whether this group consisted of heavy or light attenders to private specialist care or emergency departments of the hospitals. The immigrants in Iceland are mostly economic immigrants and not seeking asylum, so they should be in better general health than refugees coming from regions of ongoing military or political conflicts [14]. As the people attending the clinic came at random, we regard the participants as randomly selected. But with a low number of participants and with observation from a single health care centre this can be biased. We nevertheless assume that the cases represented a random group of immigrants, and the nonimmigrants are a random group of controls.

It would be interesting to analyse whether there are differences by region of origin or by the length of time immigrants have spent in Iceland. Due to the small sample size, this type of analysis was not possible. These questions could be included as future lines of research.

The strength of the study, on the other hand, lies in its design, as a case control study, with the groups evaluated being of the same age and sex and attending the same kind of service.

## 6. Conclusion

In the hope of optimizing the service of immigrants in a health care centre, in a rapidly changing society, we have tried to integrate knowledge on cultural aspects into the services as well as providing interpreters as needed. The immigrants attending the clinic came from all over the world, had similar diagnoses, and were attending the clinic as often per annum as the nonimmigrants. One-fifth of them needed translators. The health and health care utilization of immigrants did not differ from those of the nonimmigrants.

## Figures and Tables

**Table 1:** Country of origin of the immigrants.

	Number	Percent (%)
Thailand	8	14
Poland	7	12.3
Vietnam	5	8.8
Philippines	4	7
United Kingdom	3	5
Lithuania	3	5
Colombia	2	3.5
United States	2	3.5
Rumania	2	3.5
Slovakia	2	3.5

There were as well 17 other nationalities represented, one person from each country.

**Table 2:** Demographics and number of consultations at the primary health care centre. The number of consultations from the health records, found retrospectively, from the year previous to the study.

	Immigrants (%)	Controls (%)
Age^*∗*^	34 years	33.7 years
Men	9 (15.7)	9 (15.7)
Women	48 (84.2)	48 (84.2)
*Marital status*		
Single men	5 (8.7)	2 (3.5)
Single women	12 (21)	13 (22.8)
Married men	4 (7)	4 (7)
Married women^*∗∗*^	26 (45)	11 (19)
Women cohabiting	9 (15.8)	24 (42)
Unknown marital status	1 (1.7)	3 (5.2)
*Mean number of consultations the previous year*		
Personal consultations	4.6	4.7
Children's consultations^*∗∗∗*^	6.9	6.6

^*∗*^ *t*-test, *P* = 0.43;  ^*∗∗*^chi-square, *P* < 0.001;  ^*∗∗∗*^ *t*-test, *P* = 0.76.

**Table 3:** Attitudes of the immigrant and the nonimmigrant controls towards the quality of the health care service provided. Ranking of the service from 0 to 5 where 0 was unsatisfactory and 5 was the best possible service.

	On average	Significance
The nonimmigrant group	4.11	
The immigrant group	4.33	*P* = 0.27
Immigrants' rating of service in their homelands	1.7	*P* = 0.001

**Table 4:** Number of diagnoses of the participating immigrants and nonimmigrants (data from the consultations attended during the study).

	Immigrants	Controls
Muscular skeletal	8	6
Infectious	11	11
Dermatology	2	5
Depression, anxiety	1	2
Maternity care	10	10
Infant care	11	11
Cardiovascular/BP	3	1
Lungs	1	2
Administrative	3	3
Others	7	6

Total	57	57
